# Supplementary material for: Detection of Pancreatic Ductal Adenocarcinoma by Ex Vivo Magnetic Levitation of Plasma Protein-Coated Nanoparticles
Source: Cancers (Basel). 2021 Oct 14;13(20):5155. doi: 10.3390/cancers13205155 (PMC8533958; doi:10.3390/cancers13205155)
Supplement: Supplementary file 1 [file cancers-13-05155-s001.zip › cancers-1425635-supplementary.pdf]

Supplementary Materials: Detection of Pancreatic Ductal Adenocarcinoma by Ex Vivo Magnetic Levitation of Plasma Protein-Coated Nanoparticles

Luca Digiacomo, Erica Quagliarini, Vincenzo La Vaccara, Alessandro Coppola, Roberto Coppola, Damiano Caputo, Heinz Amenitsch, Barbara Sartori, Giulio Caracciolo and Daniela Pozzi

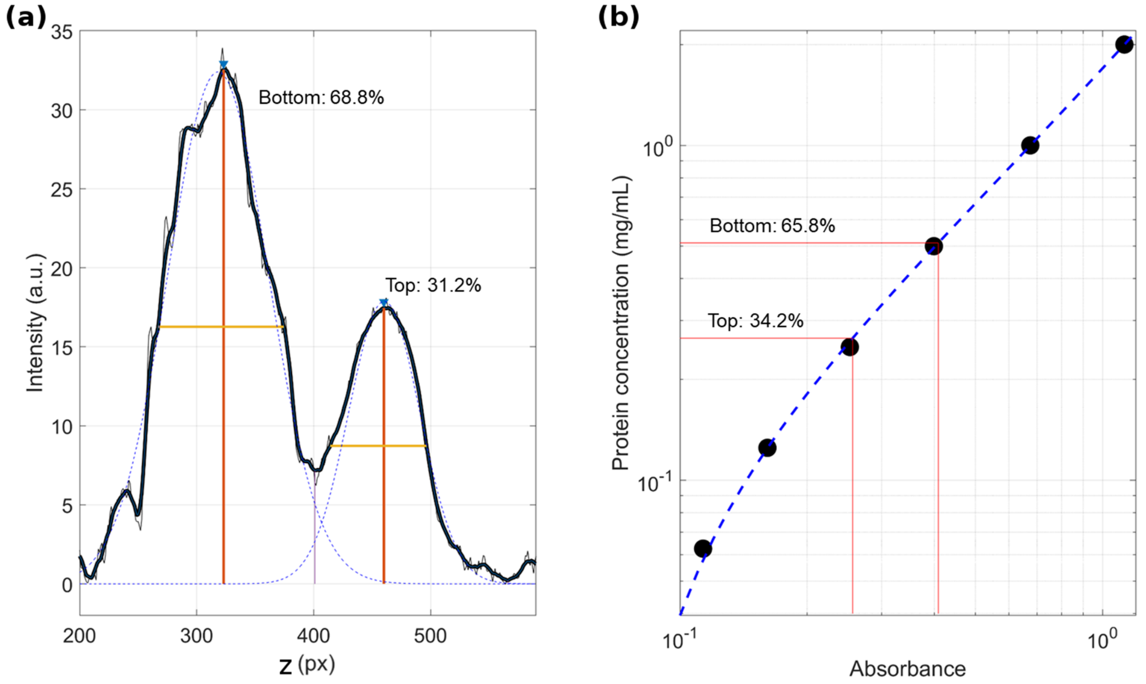

Figure S1. (**a**) Detection of peaks by image processing of MagLev patterns. Bottom and Top percentages represent the relative amount of precipitating and levitating component, respectively, computed as integral areas of the curves. (**b**) Protein content in bottom and top populations as measured by bicinchoninic acid assay (BCA). Black dots indicate the reference signal for bovine serum albumin, the blue dashed line depicts the corresponding calibration curves.

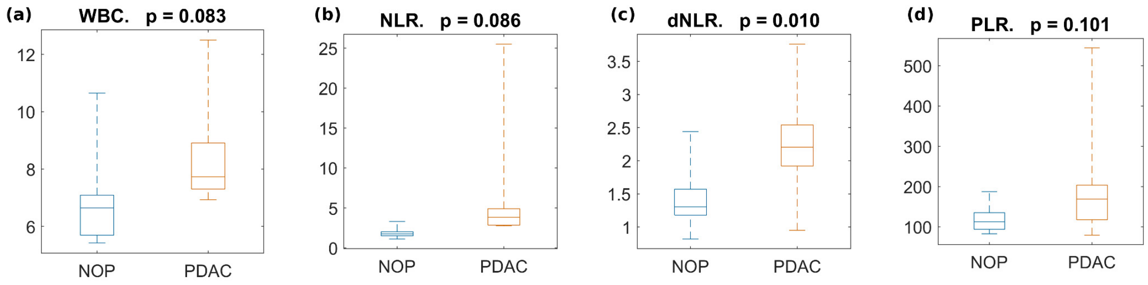

Figure S2. Distribution of systemic inflammatory response biomarkers (SIRBs). (**a**) White blood count (WBC), (**b**) neutrophils to lymphocytes ratio (NLR), (**c**) derived-NLR (d-NLR), and (**d**) platelets to lymphocytes ratio (PLR). *p*-values are evaluated using Student's *t*-test.

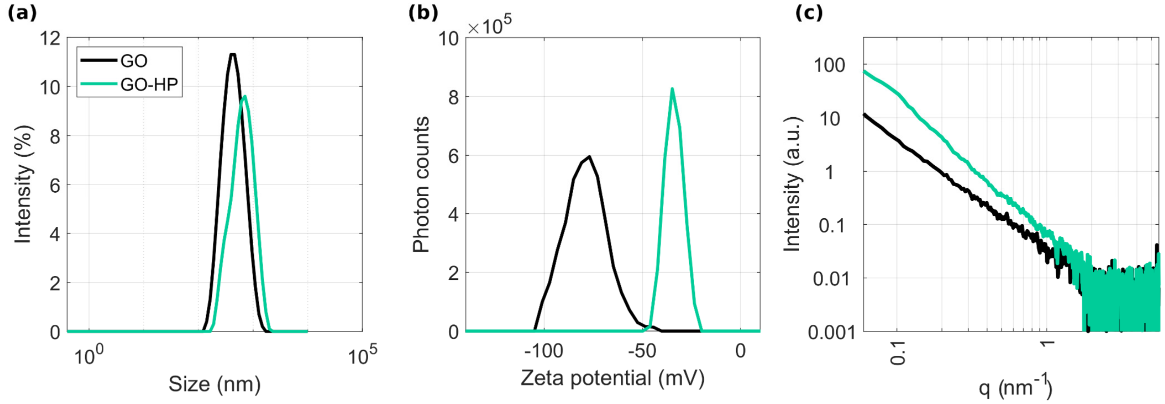

Figure S3. Characterization of GO and GO-HP complexes. (**a**) Size distributions, (**b**) zeta potential distributions, and (**c**) synchrotron small single X-ray scattering (SAXS) patterns. GO NPs were about 500 nm in size, negatively charged (zeta potential ~ −75 mV). The mass fractal dimension as determined by power law synchrotron SAXS was −2,07 (panel c, black points) that is compatible with 2D extended objects. Following exposure to human plasma (HP), average size increased, zeta potential increased, but remained negative (~ −30 mV), while power law of synchrotron SAXS patterns was −2,58. These observations are compatible with protein binding to GO NPs leading to surface decoration and small, if any, aggregation. Particle size and zeta-potential were measured at 25 °C using a Zetasizer Nano ZS90 (Malvern, U.K.). The measurements were made by diluting the sample 1:100 with distilled water and the results were reported as mean ± standard deviation of three independent replicates. Synchrotron SAXS experiments were performed at the Austrian SAXS beamline of the international synchrotron light source ELETTRA (Trieste, Italy) by using an automatic sample changer system (Haider, R., et al., µDrop: a system for high-throughput small-angle X-ray scattering measurements of microlitre samples. Journal of applied crystallography, 2021. 54(1)). Calibration of the detector (Pilatus3 1 M, Dectris, Baden, Switzerland) was done by using silver behenate powder (d-spacing = 5,8376 nm), q-range was set within 0.05 and 1.5 nm^{-1}, exposure time was 10 s (no radiation damage was observed) and temperature was controlled in the proximity of the capillary. Background subtraction, correction for primary beam intensity and detector efficiency were included in the analysis of SAXS patterns.

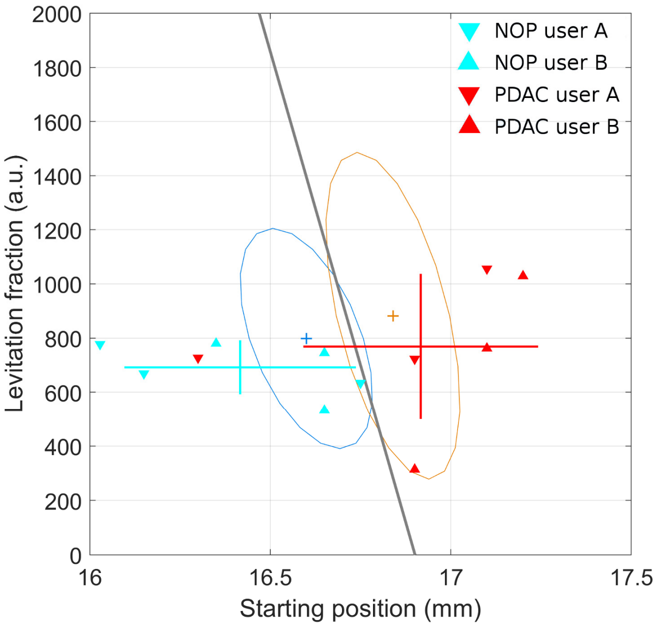

Figure S4. Inter-user reproducibility. Scatterplot of MagLev pattern's starting position and levitating population. Triangles correspond to single measurements performed for (cyan) a single NOP sample and (orange) a single PDAC sample, superimposed to the training set distributions (blue and orange) and the outcome of the linear discriminant analysis (black line), which are reported in Figure 4a.

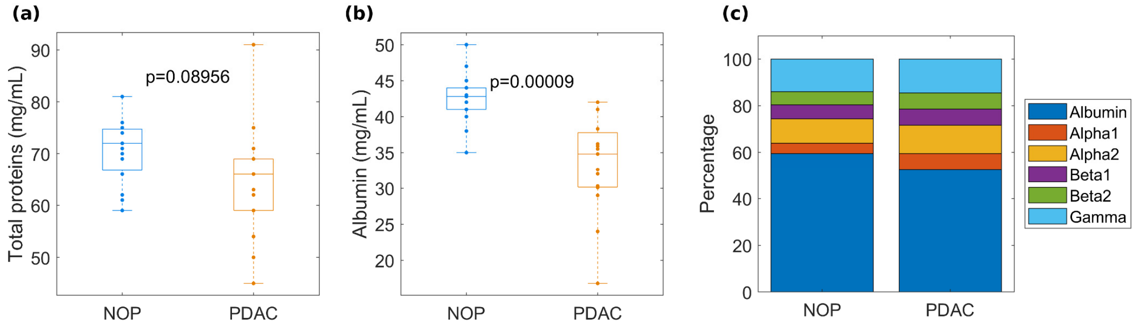

Figure S5. Protein contents. (**a**) Total protein and (**b**) albumin content and protein relative amount in plasma from NOP and PDAC subjects.

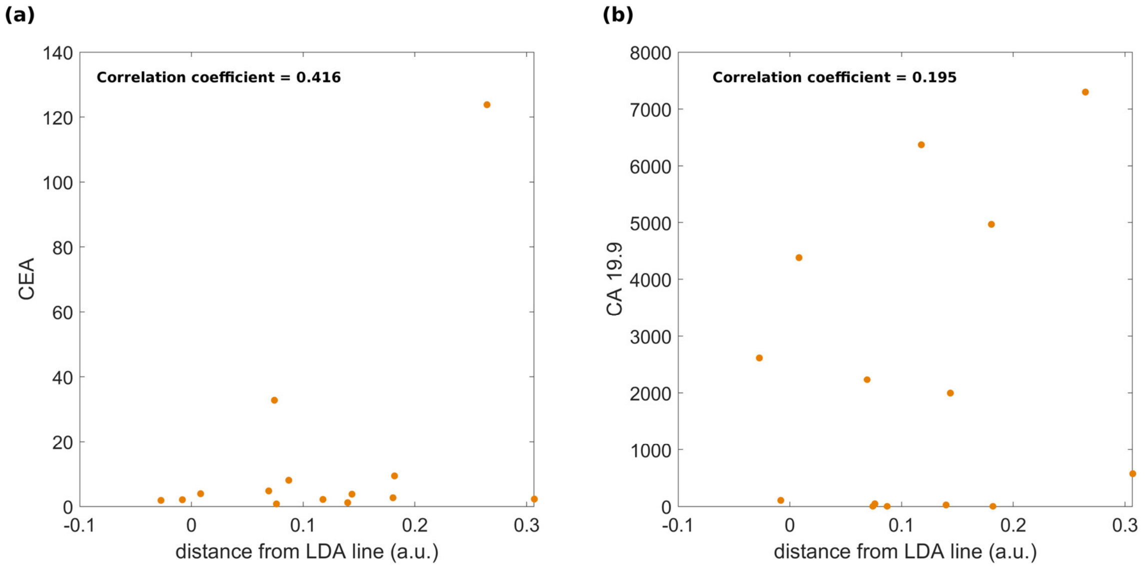

Figure S6. Correlation between CEA and CA 19.9 levels and the distance from the cut-off determined by linear discriminant analysis (LDA) reported in Figure 4a. Scatter plot of (**a**) CEA and (**b**) CA 19.9 versus the distance from the cut-off line by LDA (negative values correspond to misclassified samples) for the PDAC patients included in this study. No clear correlation was found.

Table S1. Characteristics of patients included in the training set (demographic data, American Society of Anesthesiologists Classification (ASA), Pathology).

	Healthy	*PDAC*
Sex	*N (%)*	N (%)
Female	5 (50%)	3 (30%)
Male	5 (50%)	7 (70%)
Age	*years*	*years*
Median (range) *	49.5 (23-80)	66.3 (47-79)
Median (range) in females	46.4 (25-71)	65.6 (47-79)
Median (range) in males	36.6 (23-79)	66.6 (49-75)
BMI		
Median (range)	25.1 (23.3-31.2)	25.1 (22.4-27.6)
Median (range) in females	26.6 (23.4-31.2)	25.1 (24.1-26.1)
Median (range) in males	23.6 (23.3-24.2)	25.2 (22.4-27.6)
ASA		
I	0 (0%)	0 (0%)

II	10 (100%)	3 (30%)	
III	0 (0%)	7 (70%)	
IV	0 (0%)	0 (0%)	
Pathology		*cTNM stage*	pTNM stage
Cholelithiasis	6 (60%)	IA 1 (10%)	IA 1 (10%)
Sinus pilonidalis	1 (10%)	IB 0 (0%)	IB 0 (0%)
Incisional hernia	2 (20%)	IIA 1 (10%)	IIA 1 (10%)
Inguinal hernia	1 (10%)	IIB 3 (30%)	IIB 2 (20%)
		III 4 (40%)	III 3 (30%)
		IV 1 (10%)	IV 3 (30%)

* Only Median Age was statistically significant between the two groups.

Table S2. Comorbidities of patients included in the training set.

	Healthy	*PDAC*
Hypertension	2 (20%)	2 (20%)
Ischemic heart disease	1 (10%)	1 (10%)
Obesity	1 (10%)	0 (0%)
COPD	0 (0%)	1 (10%)
Type II diabetes	0 (0%)	1 (10%)

COPD: Chronic obstructive pulmonary disease.

Table S3. Characteristics of patients included in the validation cohort (demographic data, American Society of Anesthesiologists Classification (ASA), Pathology, tumor clinical and pathological stage).

	Healthy	*PDAC*	
Sex	*N (%)*	N (%)	
Female	2 (40%)	4(80%)	
Male	3 (60%)	1 (20%)	
Age	*years*	*years*	
Median (range)	64 (42-73)	74 (59-86)	
Median (range) in females	73 (73-73)	75 (68-86)	
Median (range) in males	55 (42-64)	59	
BMI			
Median (range)	23.8 (19.5-26.1)	22.1 (18.9-24.9)	
Median (range) in females	24.5 (23-26.1)	21.3 (18.9-23)	
Median (range) in males	23.3 (19.5-25.6)	22	
ASA			
I	0 (0%)	1 (20%)	
II	5 (100%)	4 (80%)	
III	0 (0%)	0 (0%)	
IV	0 (0%)	0 (0%)	
Pathology		*cTNM stage*	pTNM stage
Cholelithiasis	2 (40%)	IA 0 (0%)	IA 0 (0%)
Diverticulosis	1 (20%)	IB 0 (0%)	IB 0 (0%)
Inguinal hernia	1 (20%)	IIA 0 (0%)	IIA 0 (0%)
Hiatal hernia	1 (20%)	IIB 0 (0%)	IIB 0 (0%)
		III 2 (40%)	III 0 (0%)
		IV 3 (60%)	IV 5 (100%)

No statistically difference were observed in the two groups.

Table S4. Comorbidities of patients included in the validation cohort.

	Healthy	*PDAC*
Hypertension	1 (20%)	2 (40%)
Ischemic heart disease	1 (20%)	1 (20%)
Gastroesophageal reflux disease	1 (20%)	0 (0%)
Dyslipidemia	0 (0%)	1 (20%)

Table S5. Inter-user reproducibility. *p*-values from Student's *t*-test evaluated over two triplicates for a NOP sample and two triplicates for a PDAC sample. All the *p*-values are much larger than the threshold commonly used for the evaluation of the statistical significance, i.e. $p = 0.05$. Therefore, no statistical significance was found between the measurements obtained by two independent users.

	inter-user *p*-value from Student's *t*-test			
	starting position	speed	precipitating fraction	levitating fraction
NOP	0.364	0.446	0.222	0.899
PDAC	0.306	0.863	0.255	0.601
